# Hypernatremia: Correction Rate and Hemodialysis

**DOI:** 10.1155/2014/736073

**Published:** 2014-11-09

**Authors:** Saima Nur, Yasir Khan, Saadia Nur, Hassan Boroujerdi

**Affiliations:** ^1^Wellesley College, 106 Central Street, Wellesley, MA 02481, USA; ^2^Department of Internal Medicine, University of Arizona, 1501 N. Campbell Avenue, Tucson, AZ 85724, USA; ^3^University of California Los Angeles, Los Angeles, CA 90095, USA; ^4^Department of Nephrology, Yale-New Haven Hospital, P.O. Box 208029, New Haven, CT 06520, USA

## Abstract

Severe hypernatremia is defined as serum sodium levels above 152 mEq/L, with a mortality rate ≥60%. 85-year-old gentleman was brought to the emergency room with altered level of consciousness after refusing to eat for a week at a skilled nursing facility. On admission patient was nonverbal with stable vital signs and was responsive only to painful stimuli. Laboratory evaluation was significant for serum sodium of 188 mmol/L and water deficit of 12.0 L. Patient was admitted to medicine intensive care unit and after inadequate response to suboptimal fluid repletion, hemodialysis was used to correct hypernatremia. Within the first fourteen hours, sodium concentration only changed 1 mEq/L with a fluid repletion; however, the concentration dropped greater than 20 mEq/L within two hours during hemodialysis. Despite such a drastic drop in sodium concentration, patient did not develop any neurological sequela and was at baseline mental status at the time of discharge.

## 1. Introduction

Hypernatremia is a relatively common electrolyte abnormality. It occurs most often in extremes of ages—in pediatric and geriatric populations—as members of both are less able to express their thirst and are often fed high-solute formulations that cannot be managed by their renal concentrating abilities [1, 2]. Aging physiology places geriatric population at the highest risk for developing hypernatremia. With advancing age, thirst is decreased and the ability to dilute and concentrate urine during water loading and deprivation diminishes [3]. In addition, the glomerular filtration rate falls and total body weight decreases. Consequently, the loss of even small amounts of water leads to a rather pronounced development of hypernatremia in an elderly person [3].

Regardless of the underlying cause of hypernatremia, serum sodium levels above 160 mEq/L are associated with very high mortality rates—even 100%—if the levels are not corrected within 10 days of onset [3, 4]. Literature provides supporting and counter evidence regarding faster correction rate in hypernatremia [5, 6]. However, it is noteworthy that persistent hypernatremia is associated with a much higher mortality [6]. Current standard recommendations are to lower serum sodium levels in hypernatremic patients slowly, as rapidly lowering serum sodium places patients at a higher risk of developing cerebral edema [6, 7]. Most clinicians recommend correction rate below 0.5 mEq/L/hr and at most drop 10–12 mEq/L in 24 hrs for patients with hypernatremia, unless hypernatremia has developed in few hours [8]. We present a case in which an elderly nursing home patient had a serum sodium level of 188 mEq/L that was treated with hemodialysis, unexpectedly, at a faster correction rate, after an inadequate response with fluids. Patient did not develop any neurological sequela and reached base line mental status before discharge.

## 2. Case Presentation

An 85-year-old gentleman with past medical history significant for hypertension, congestive heart failure, coronary artery disease, mild dementia, and depression was brought into Whittier Medical Center emergency room for an altered level of consciousness. One month prior to admission, patient had undergone hemiarthroplasty of the left hip and was transferred to a skilled nursing facility for physical therapy.

As per nursing home staff, patient had stopped eating and drinking for a week. In emergency room patient was nonverbal and was responsive only to painful stimuli but with stable vital signs (BP103/69, HR88, RR18, and T98.2F). Physical exam was significant for dry mucous membrane and poor skin turgor and diminished breath sounds at both bases, most likely due to decreased respiratory effort with altered mental status. Cardiac and abdominal examinations were unremarkable. Patient had intact bilateral corneal reflex and Babinski was absent bilaterally and there was no sign of peripheral edema.

## 3. Investigations

Laboratory evaluation showed following values: hematocrit 40.0%, WBC count 11.0 with normal differential cell count, serum sodium 187 mmol/L, potassium 4.6 mmol/L, chloride 142 mmol/L, and CO_2_ of 23.0 mmol/L. Glucose concentration was 7.05 mmol/L, BUN level was 70.68 mmol/L, and creatinine was 0.627 mmol/L (BUN/Cr, 112.19/1). Calcium level was 2.23 mmol/L, troponin was 0.13 *μ*g/L, and amylase and lipase were 179 and 324 U/L with lactic acid at 1.4 mmol/L and BNP at 536 ng/L. Urine analysis showed specific gravity 1.020, urine output of 340 mL the first day and urine osmolality of 532 mOsm/K, pH5.0 without red or white blood cells. EKG demonstrated sinus rhythm with right bundle branch block and nonspecific ST-T abnormalities and echocardiogram revealed normal ejection fraction without any wall motion abnormalities. Head CT was negative for subdural hematoma, hydrocephalus, or structural changes and chest X-ray and renal ultrasound were also unremarkable. Additionally, patient was not on any diuretics.

## 4. Differential Diagnosis

Differential diagnosis considered for altered mental status included structural brain pathology (stroke/subdural hematoma/hydrocephalus), infection (UTI, pneumonia, and wound), postoperative confused state compounded with pain medications, electrolyte abnormalities (sodium, calcium, and magnesium), and endocrine causes and toxins. Upon diagnosis of hypernatremia, differentials considered for patient's hypovolemic hypernatremia included decreased water intake versus increased water losses (diuretics, osmotic diuresis, renal failure, diarrhea, and respiratory losses).

## 5. Treatment

Patient was admitted to the medicine intensive care unit, where intravenous fluids (1/2 NS at 100 mL/hr) were rapidly administered for dehydration and hypernatremia; serial monitoring of electrolytes and osmolality was also initiated. The estimated water deficit with Adrogue's formula [water deficit = total body water × ((serum Na/140) − 1)] was calculated to be 12.0 liters [6]. Patient was maintained on 1/2 NS at 100 mL/hr and underwent dialysis four times in the ICU.  Table 1 lists dialysis dates, duration, serum sodium, BUN/Cr, fluid balance, and dialysate sodium and blood flow and dialysate flow rates. Similarly, Table 2 lists change in serum sodium and urea over three treatment days in the ICU. Within the first fourteen hours, with IVF, there was only change of 1 unit in serum sodium. Although, fluid replacement rate should have been increased, it was deemed appropriate to lower serum sodium with hemodialysis, as persistent severe hypernatremia in this patient was felt to be incompatible with life. However, two hours into dialysis serum sodium concentration dropped 21 units, but for the rest of the treatment correction rate remained below 1 mEq/L/hr.

## 6. Outcome and Follow-Up

Patient's renal function improved, with urea/Cr dropping to 61.69 from 112.19, and urine output increased as well. After four days, patient was discharged from Whittier Medical Center ICU to Presbyterian Intercommunity Hospital Medicine Ward, where serum sodium levels remained within normal range and he was discharged to a skilled nursing facility after 12 days. At follow-up visit in the office after two weeks patient was interactive and per family was at his baseline.

## 7. Discussion

Hypernatremia is classified as serum sodium levels above 145 mEq/L [4]. It develops from two primary mechanisms: excess water loss and excess solute gain [9]. Our patient was severely dehydrated secondary to diminished repletion of fluids, as he had refused to eat or drink for a week. Over the course of a week, patient became severely altered and was semicomatose at the time of admission. Hypernatremia initially causes fluid movement out of the brain that leads to cerebral contraction and consequently manifests as altered mental status. The brain then responds to hypernatremia in two phases to counter cerebral contraction. In the first stage, an acutely rapid uptake of electrolytes helps counter decreasing cerebral volume. In the next stage, the slower accumulation of organic osmolytes attempts to maintain a steady brain volume [9–11]. When correcting hypernatremia, one must be aware of these acute and chronic changes, as major differences in serum osmolality can cause dramatic shifts in water movement, eventually leading to cerebral edema manifesting as seizures, permanent neurologic damage, or even death [3, 7–9].

Recently, Lima et al. published a teaching case highlighting management guidelines for chronic hypernatremia with emphasis on slower correction rate [8]. On the other hand, Alshayeb et al. published a clinical investigation highlighting the association of persistent hypernatremia with increased mortality [4]. Patients with chronic hypernatremia underwent slower correction rate and remained hypernatremic for several days, thereby contributing to the increased mortality in chronic hypernatremic group [4]. This puts clinicians in a dilemma as to what is a best method for correcting hypernatremia in a severely hypernatremic patient, with sodium concentration near 200 mEq/L. A faster correction rate has associated risk of cerebral edema but a slower correction rate promotes persistent hypernatremia. Both of these scenarios are associated with increased mortality.

We realize that true physiology dictates slower correction rates in chronic stages and faster rates in acute stages. The goal in managing hypernatremia is to correct the water deficit in a reasonable time frame while avoiding untoward side effects [6–8, 10]. In patients with chronic hypernatremia, it is recommended that correction occur over a period of 2 to 3 days with a maximum serum sodium correction rate of 0.5 mEq/L/hr or a decrease in serum sodium level of 10 to 12 mEq/Lin a 24 hr period [4, 6–8]. However, no prospective studies completely validate such recommendations [4, 8]. Previously, Snyder et al. have illustrated that mortality is higher with faster correction rate, but recovery has also been observed in few cases with a faster correction rate [6]. Hemodialysis has also been used for treatment of acute hypernatremia after observing failure with conventional methods [4]. However, current management guidelines do not comment on effective use of dialysis for management of hypernatremia [2, 8].

Hemodialysis has advantages over conventional methods as it allows quick removal of excess sodium and fluid repletion. Furthermore, dialysis allows better control over fluid and electrolytes in situations complicated by cardiopulmonary or renal disorders [8]. Unfortunately, correction rates seem to be autonomous during hemodialysis [12], but this approach avoids serious conditions that can occur with persistent hypernatremia [4]. We believe that hemodialysis can play a very beneficial role in management of acute severe hypernatremia. For instance, a patient with serum sodium levels near 200 will remain hypernatremic for five days, if treated with conventional fluid replacement methods, but with help of dialysis, theoretically, this period can be curtailed. We were worried about the fast correction rate in our patient, as it was not clear whether this was an acute or chronic hypernatremia but his serum sodium level was certainly incompatible with life. We used the maximum sodium dialysate for dialysis; however, sodium concentration still dropped 21 units in 2 hours. Several cases of hypernatremia have been treated with hemodialysis but in most of them sodium decreased by 19 to 20 mEq/L within four hours of dialysis [12]. Fortunately, no neurological complications were observed in any of these patients [12]. It is not clear if rapid correction of hypernatremia with hemodialysis causes neurologic complications as few cases of hypernatremia with hemodialysis have been reported without any neurological complications despite the faster correction rate [13]. Likewise, our patient did not develop any neurological complication despite such high correction rate. Theoretically, blood flow rate and dialysate flow rate can be manipulated to achieve the desired correction rate; however, in previously reported cases, with the maximum concentration of dialysate, correction rate could not be predicted [14]. A safer approach in chronic hypernatremic patient would be continuous renal replacement therapy where sodium levels can be drawn every hour and fluid rate in addition to dialysate concentration can be changed easily.

To be noted, without further research this method should only be considered in complicated cases where extremely severe hypernatremia levels are incompatible with life. We need to stress the fact that our approach is investigational and should be reserved for very unusual circumstances. Sodium concentration of 190 mEq/L is incompatible with life; thus, our primary goal for our patient was to bring sodium concentration to levels compatible with life and then treat hypernatremia. We realize that initially patient was treated with suboptimal correction rate but with such high sodium levels we chose hemodialysis to bring sodium to a level compatible with life.

Our case, along those of Park and Alshayeb, makes one curious about the significance of correction rate in severely hypernatremic patients, especially with hemodialysis. It is not clear why, despite such high correction rate with hemodialysis, neurological complications are not observed. We want to stress the fact that we are not attempting to change the conventional thinking, but perhaps this report can bring focus to the role of hemodialysis in management of severely hypernatremic patients resistant to conventional therapy.

## Figures and Tables

**Table 1:** Dialysis and fluid balance.

Date	Duration	Na (D)	Na (S)	Bun/Cr	Fluid balance
3/28/2012	4	150	190	198/6.09	1050
3/29/2012	1.2	140	161	97/3.80	N/A
3/30/2012	3	135	150	69/3.24	N/A
4/2/2012	3	135	139	47/3.49	−2000

Total IVF: 1440 mL due to infusion rate of 100 mL/hr for 6 days					
Bolus IVF 500 on 3/30/12					
Dialysis: 2000 − 1050 = 950					
Blood flow rate: 250 mL/min; dialysate flow rate: 600 mL/min					
Total IVF: 13950 mL					

**Table 2:** 

Time	Sodium level	Time-change	Concentration-change	Osmolality-calculated	Urea mmol/L
03/27/12—2255	187 mEq/L			451.8	70.69
03/28/12—0230	190 mEq/L	4 hrs	+3	456.5	70.69
03/28/12—1253	188 mEq/L	10 hrs	−2	451.6	70.33
03/28/12—1513	167 mEq/L	2 hrs	−21	374.6	34.99
03/28/12—1820	161 mEq/L	3 hrs	−6	357	29.63
03/28/12—2015	161 mEq/L	2 hrs	0	356.9	29.99
03/29/12—0530	161 mEq/L	9 hrs	0	362.4	34.63
03/30/12—0445	151 mEq/L	24 hrs	−10	334.3	24.63
03/31/12—0505	144 mEq/L	24 hrs	−7	304.6	11.78
